# Prevalence of Zinc Deficiency in Inflammatory Bowel Disease: A Systematic Review and Meta-Analysis

**DOI:** 10.3390/nu14194052

**Published:** 2022-09-29

**Authors:** Roberta Zupo, Annamaria Sila, Fabio Castellana, Roberto Bringiotti, Margherita Curlo, Giovanni De Pergola, Sara De Nucci, Gianluigi Giannelli, Mauro Mastronardi, Rodolfo Sardone

**Affiliations:** 1 Unit of Data Sciences and Technology Innovation for Population Health, National Institute of Gastroenterology “Saverio de Bellis”, Research Hospital, 70013 Castellana Grotte, Italy; 2 U.O.S.D. Digestive Endoscopy Ospedale Di Venere, 70131 Bari, Italy; 3 Section of Gastroenterology II, National Institute of Research “Saverio De Bellis”, 70013 Castellana Grotte, Italy; 4 Unit of Geriatrics and Internal Medicine, National Institute of Gastroenterology “Saverio de Bellis”, Research Hospital, 70013 Castellana Grotte, Italy; 5 Scientific Direction, National Institute of Research “Saverio De Bellis”, 70013 Castellana Grotte, Italy

**Keywords:** zinc deficiency, inflammatory bowel disease, meta-analysis

## Abstract

Malabsorptive disorders are closely associated with micronutrient deficiencies. In inflammatory bowel disease (IBD), trace element deficiencies pose a clinical burden from disease onset throughout its course, contributing to morbidity and poor quality of life. We aimed to conduct a systematic review and meta-analysis of the prevalence of zinc deficiency in IBD. Literature screening was performed on six electronic databases until 1 May 2022. Two independent investigators assessed the 152 retrieved articles for inclusion criteria, met by only nine, that included 17 prevalence entries for Crohn’s disease (CD) (*n* = 9) and ulcerative colitis (UC) (*n* = 8). No exclusion criteria were applied to language, deficiency cut-offs, population age, general health status, country, or study setting (cohort or cross-sectional). The prevalence of zinc deficiency in blood was scored positive if due to a single disease, not cumulative factors. Zinc deficiency prevalence across selected studies showed higher values in CD than in UC. Pooled analyses by the IBD subgroup showed a total population of 1677 with CD, for an overall mean zinc deficiency prevalence of 54% and 95% confidence intervals (CI) ranging from 0.51 to 0.56, versus 41% (95%CI 0.38–0.45) in the UC population (*n* = 806). The overall prevalence at meta-analysis was estimated at 50% (95%CI 0.48–0.52), but with high heterogeneity, *I*^2^ = 96%. The funnel plot analysis failed to show any evidence of publication bias. The risk of bias across selected studies was moderate to low. In IBD contexts, one of two patients suffers from zinc deficiency. Mismanagement of micronutrient deficiencies plays a role in inflammation trajectories and related cross-pathways. Clinicians in the field are advised to list zinc among trace elements to be monitored in serum.

## 1. Introduction

Zinc is among the trace inorganics that are found in body fluids and tissues in small amounts but are essential for body growth and function. About 85% of zinc in the body is found in muscle and bone, 11% in skin and liver, and the rest in all other tissues. Interestingly, no single test reflects the zinc status in the whole body; however, tests for plasma or serum zinc are the most widely used [1]. Zinc in plasma is bound nearly 60% to albumin, 40% to macroglobulins, and 3% to amino acids and the renal ultrafiltration fraction [2]. Human metabolic pathways show that zinc is involved in the function of many enzymes, being an integral component of nearly 10 percent of the human proteome (e.g., of several key enzymes and transcription factors). According to recent dietary guidelines, an adult daily intake of 11 mg (males) and 8 mg (females) is recommended [2]. Dietary zinc sources include a wide range of edible sources [3]. Oysters have the greatest zinc concentration per serving, although red meat and poultry supply most of the zinc in the diet. Beans, nuts, different types of shellfish (such as crabs and lobsters), whole grains, fortified breakfast cereals, and dairy products are other good sources [4]. Phytates, found in whole-grain bread, cereals, legumes, and other foods, bind to zinc and prevent its absorption [5,6]. Consequently, zinc bioavailability from cereal and plant diets is lower than from animal foods, despite the significant zinc content in many cereal and plant foods. Citric acid may improve absorption, whereas iron, copper, calcium, fiber, and phytates may inhibit it. Zinc is an essential element for the integrity of bodily structures and activities. Zinc acts as a cofactor for various enzymes involved in growth, cell signaling pathways, cellular activities, immune function, and tissue repair.

Once ingested, zinc is absorbed in the small intestine, both the distal duodenum and proximal jejunum. However, research has yet to shed light on zinc homeostasis in enterocytes and the molecular processes intrinsic to intestinal absorption. In particular, the transfer of zinc through enterocytes upon absorption, its subsequent basolateral release into the bloodstream, and the involvement of zinc-binding or zinc-transport proteins in this process need to be elucidated, apart from the already known metallothionein. In addition, the involvement of zinc-transporters in the cytoplasmic organelles of enterocytes (such as ZnT-2, ZnT-4, ZnT-6, and ZnT-7) in cellular zinc trafficking and homeostasis needs to be investigated in intestinal cell models in vitro to understand the regulation of zinc transit at the enterocyte level. Zinc levels are often low in patients with chronic diarrhea and malabsorptive disorders [7].

This is why trace elements deficiency is common in patients with inflammatory bowel disease (IBD) during both active disease and remission [8,9]. Increased zinc losses occur mostly in conjunction with diarrhea, ostomies, and high-exit fistulas, often experienced in IBD. In conjunction with the chronic malabsorption state in cases of intestinal inflammation, micronutrient leaks are likely responsible for zinc deficiency from the disease onset. Reports indicate that subclinical zinc deficiency may lead to mucosal inflammation in these patients, as well as exacerbate colitis, and increase the production of pro-inflammatory cytokines [10]. 

Of note, biologically speaking, zinc homeostasis is strongly affected by a balance between the zinc-binding protein metallothionein and the expression of two zinc transporters. Because albumin is the zinc transporter, a low albumin level, mainly common to IBD patients experiencing malnutrition, malabsorption, an increased fractional catabolic rate of albumin, and increased albumin transfer out of the vascular system, may affect zinc levels.

Extensive reports so far substantiate the burden of micronutrient deficiencies in malabsorptive settings. However, studies investigating zinc deficiency in IBD patients are few, heterogeneous, and were performed in small patient subsets. The findings are often fragmented, whereas the deficiencies spectrum is broad. Here, we conducted a systematic review and meta-analysis of available data to estimate the prevalence of zinc deficiency in IBD, looking at the pattern of prevalence profiles across the two well-known forms of IBD, presumed to reflect the intrinsic difference in the inflammatory site.

## 2. Methods

### 2.1. Search Strategy, Selection Criteria, and Data Extraction

A computerized literature search of MEDLINE and the Cochrane database did not identify any previous systematic reviews on the prevalence of zinc deficiency in IBD. The present systematic review followed the Preferred Reporting Items for Systematic reviews and Meta-Analyses (PRISMA) guidelines, adhering to the PRISMA 27-item checklist [11]. An *a priori* protocol for the search strategy and inclusion criteria was established and recorded, with no particular changes to the information provided at registration on PROSPERO, a prospective international registry of systematic reviews (CRD42023330824). We performed separate searches in the US National Library of Medicine (PubMed), Medical Literature Analysis and Retrieval System Online (MEDLINE), EMBASE, Scopus, Ovid, and Google Scholar to retrieve original articles investigating serum zinc levels and the prevalence of zinc deficiency in IBD populations. The primary objective was to assess a pooled prevalence of a plasma zinc concentration deficit in IBD settings. We also considered the gray literature using the massive preprint archive https://arxiv.org/in (accessed on 1 August 2020) in the study selection phase, and the database http://www.opengrey.eu/to (accessed on 1 August 2020) to access notable conference abstracts and other non-peer-reviewed material. No exclusion criteria were applied to language, the defined deficiency status cut-off, nor population age, general health status, country, recruitment settings (hospital, community, or home care), and study setting (trials, cohort, or cross-sectional). We used only original articles investigating IBD populations and providing disease-specific prevalence data separately for Crohn’s and ulcerative colitis, as an inclusion criterion.

The research strategy used in PubMed and MEDLINE and adapted to the other four electronic sources included the keywords “zinc”, “inflammatory bowel disease”, “Crohn’s disease”, and “ulcerative colitis” combined through the use of Boolean indicators such as “AND” and “OR”. The search strategy used the Boolean indicator “NOT” to rule out letters, revisions, and meta-analyses. The literature search had no time restriction, and papers were retrieved until 1 May 2022. No language restrictions were made. Two researchers (RZ, AS) conducted the searches, reviewed titles and abstracts of articles retrieved separately and in duplicate, checked full texts, and selected the papers for inclusion in the study. Technical reports, letters to the editor, and systematic and narrative review articles were excluded. Inter-rater reliability (IRR) was used to estimate inter-coder agreement and the κ statistic to measure accuracy and precision. In accordance with PRISMA concepts and the quality assessment steps, a coefficient k of at least 0.9 was obtained in all data extraction steps [12].

### 2.2. Data Analysis

Two investigators (RZ, AS) extracted the following information separately and in duplicate in piloted form: author, publication year, survey year, country, and design (longitudinal, cross-sectional). Researchers tabulated data by IBD type of disease (Crohn’s disease, CD, and ulcerative colitis, UC) to retrieve information on (1) sample size (*n*), (2) age (expressed as mean ± standard deviation, SD, or interquartile range, IQR), (3) male and female representativeness (expressed as *n* and %), serum zinc levels (according to IBD type, where possible, and expressed as mean ± SD or median and IQR), (4) threshold value used to assess zinc deficiency, (5) prevalence of zinc deficiency by IBD, and (6) summary of study findings. All references selected for retrieval from the databases were managed with the MS Excel data collection software platform by an expert biostatistician (FC). Finally, the data extracted from the selected studies and stored in the database were structured as evidence tables.

**Figure 1:**
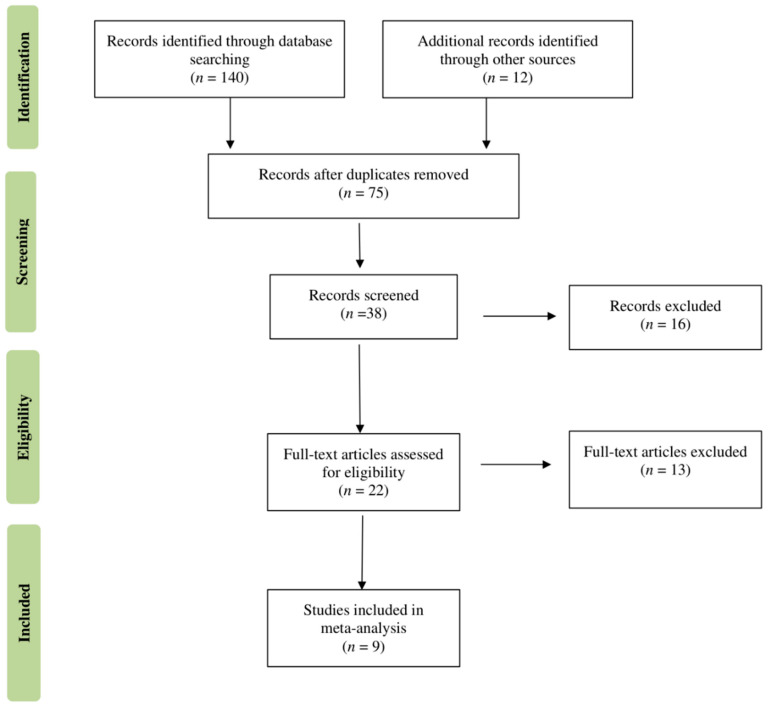
Flow diagram of literature screening process.

**Figure 2:**
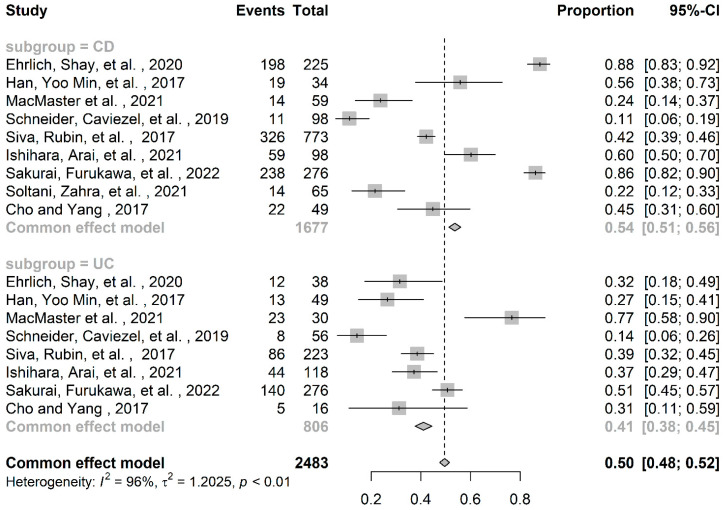
Pooled and grouped prevalence of zinc deficiency in IBD [15,16,17,18,19,20,21,22,23].

**Figure 3:**
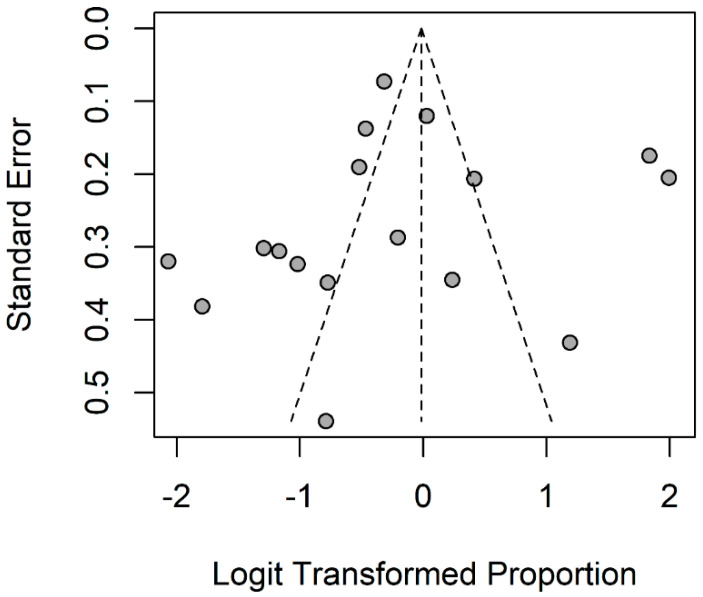
Funnel plot for assessment of publication bias across selected studies. (*n* = 9).

The quality of the studies included in the meta-analysis was evaluated using a tool developed by Hoy and colleagues [13]. Each study was assigned a score of one (yes) or zero (no) for each of the ten criteria. Based on the total score, studies were categorized as having a low (>8), moderate (6–8), or high (≤5) risk of bias. Disagreements between the two investigators on the methodological quality of the included studies were addressed by discussion, involving a third investigator in the final agreement (RS). All data analyses were performed using R, version 2021.09.1; our biostatistician (FC) used the meta-package to conduct meta-analyses of the zinc deficiency prevalence (%), subdivided according to IBD illness type (CD, UC). A common-effects model was used to calculate the prevalence and 95% confidence intervals (CI) (Figure 1 and Figure 2). The Higgins and colleagues [14] *I*^2^ test was used to estimate percentage heterogeneity between studies that cannot be explained by chance. The closer this value is to zero, the less the variability between studies. Negative values are comparable to zero and indicate that there is no heterogeneity. Values below 25% suggest a low, between 25% and 50% moderate, and above 50% high heterogeneity among studies. The funnel plot shown in Figure 3 was used as a visual tool when investigating publication bias. The horizontal axis shows the scatter of treatment effects estimated from individual studies, while the vertical axis shows study size.

## 3. Results

The first systematic search of the literature yielded 152 entries. After excluding duplicates, 75 were classified as potentially relevant and selected for the title and abstract analysis. Then, 37 were excluded for failure to meet the characteristics of the approach or the review goal. After reviewing the full text of the remaining 38 records, only 9 met the inclusion criteria and were included in the meta-analysis [15,16,17,18,19,20,21,22,23]. The Preferred Reporting Items for Systematic Reviews and Meta-analyses (PRISMA) flow chart illustrating the number of studies at each stage of the review is shown in Figure 1. The final study base included nine articles reporting zinc deficiency prevalence by IBD condition (CD, UC). 

Details of the design (cohort or cross-sectional), sample size (*n*) and gender ratio (%), survey year, study population, age range, serum zinc levels at recruitment, zinc deficiency cutoffs, country, and summary of findings are provided in Table 1. The cross-sectional design (67%, *n* = 6) predominated over the longitudinal (33%, *n* = 3). Recruitment settings were all community-based, and the geographic distribution of studies favored Asia (67%, *n* = 6), followed by Europe (22%, *n* = 2) and America (11%, *n* = 1). Following the inclusion criteria, all subjects had IBD. Only one study investigated subjects with Crohn’s disease alone, whereas the other eight analyzed both IBD conditions. In total, this meta-analysis analyzed 736 subjects suffering from UC and 1677 with CD, resulting in a total IBD population of 2413 subjects. About 46% of the CD population and ~47% of the UC population were female. The prevalence of zinc deficiency across the selected studies showed higher mean values in the CD than in the UC population. Clustered analyses by IBD subgroup (CD, UC) showed a total population of 1677 for CD, with an overall zinc deficiency mean prevalence of 54% (95%CI 0.51 to 0.56). Notably, within this subgroup, the largest reports by Ehrlich and colleagues [15] and Sakurai and colleagues [21] reported a higher prevalence of 88% (95%CI 0.83 to 0.92) and 86% (95%CI 0.82 to 0.90), respectively (Table 2). In contrast, the total UC population of 806 subjects showed an overall 41% prevalence (95%CI 0.38 to 0.45) of zinc deficiency. The prevalence was more evenly distributed within this subgroup, apart from in the MacMaster and colleagues study [17], which reported significantly higher numbers (77%, 95%CI 0.58 to 0.90). The meta-analysis produced an overall estimate of 50% (95%CI 0.48 to 0.52) zinc deficiency prevalence, with high heterogeneity *I*^2^ = 96% (Table 2). The funnel plot analysis showed no evidence of publication bias (Figure 2). According to the 10-item quality assessment checklist for prevalence studies by Hoy and colleagues [13], we found a moderate (*n* = 5) [16,17,18,21,22] to low (*n* = 4) [15,19,20,23] risk of bias across selected studies (Table 1). 

## 4. Discussion

The present systematic review and meta-analysis aimed to provide a revised estimate of the prevalence of zinc deficiency in IBD populations, without restrictions as to the country, patients age, and study design. We clustered the 17 entries from the nine studies conducted in three different countries (Asia, Europe, and America) that had reported single prevalence data by type of IBD within the population examined. To the best of our knowledge, no meta-analytic report has yet been published in the literature on this topic. Given the growing concern about the management of individuals with chronic malabsorption diseases, zinc deficiency in CD and UC is among the major sensitive issues. As the main finding, this meta-analysis produced an overall zinc deficiency estimate of 50% (95%CI 0.48–0.52), with high heterogeneity *I*^2^ = 96% and a moderate to low risk of bias across selected reports. Funnel plot analysis showed no evidence of publication bias. The clustered meta-analysis by the IBD group (CD and UC) showed a higher overall prevalence of zinc deficiency in the CD group than in the UC group (54%, 95%CI 0.51 to 0.56, versus 41%, 95%CI 0.38 to 0.45). In fact, while CD is known to affect any part of the gastrointestinal tract, including the mouth, esophagus, stomach, small and large intestines, rectum, and anus, UC compromises the colon and rectum. Moreover, the more malabsorptive CD affects all layers of the intestinal wall discontinuously, whereas in UC, the inflammation occurs in the innermost lining of the intestinal wall and is a continuous stretch within the colon.

Of the trace element deficiencies so far reported in the literature, zinc deficiency in IBD patients may result from poor oral intake and especially from the intrinsic malabsorptive nature of IBD. As proven by preclinical models and translational studies in humans, the relationship between trace zinc and chronic malabsorptive disease must be considered bidirectional since low serum zinc concentrations may also exacerbate inflammation through dysfunction and deficient epithelial barrier reconstruction, altered mucosal immunity, and increased pro-inflammatory cytokines [24,25,26]. In support of these mechanistic hypotheses, a recent report suggested that zinc supplementation might favor permeability modifications in CD patients in remission. Thus, an improved intestinal barrier function may help reduce the recurrence risk, especially in CD [9]. Indeed, the enhanced gastrointestinal epithelial barrier function driven by zinc may play an essential role In potential therapeutic actions, especially in CD, which is more malabsorptive than UC. This latter point is also corroborated by the known proximal intestinal sites of zinc absorption, i.e., the duodenum and jejunum [27].

Furthermore, previous research suggested that zinc may have some efficacy in modulating the immune system through an improved response to pathogens, reduced inflammatory response, and improved atopic/allergic reactions. Zinc is also involved in cell cycle regulation, particularly apoptosis, and hence has potential anticarcinogenic effects. All these effects have a “symbiotic” relationship with the gut microbiota [28]. From a prognostic perspective, zinc deficiency may predispose to growth retardation in young populations, and to loss of appetite, impaired immune function, and structural impairment of the intestinal endothelium. In severe cases, it may also drive hair loss, diarrhea, delayed sexual maturation, impotence, hypogonadism in males, and eye and skin lesions [29]. All these aspects highlight the importance of early nutritional preventive management in IBD settings, from better quality of life and healthcare burden perspectives. As to the loss of appetite reported in zinc deficiency [30], lower trace zinc values may also facilitate unintentional weight loss, malnutrition [31], sarcopenia, and cachectic states, mainly in restrictive dietary settings. With regard to the structural impairment of the intestinal endothelium, intervention studies on CD patients observed that zinc supplementation has some potential to reduce transmucosal leakage in these patients [9]. This latter finding is critical because intestinal epithelial barrier dysfunction may allow leukocytes to pass through, causing exposure to a “storm” of luminal antigens, a hallmark of IBD activity. Therefore, zinc supplementation may reduce the inflammatory response and maintain remission in CD. However, this empirical evidence is not yet supported by specific guidelines regulating supplementation [32]. 

In order to supplement the biological explanation of our prevalence data, we looked at information on inflammation in the selected reports, considering CRP values and disease activity scores in relation to zinc deficiency. On the one hand, we found considerable inconsistency in the data, as a minority of 33% indicated a significant correlation between zinc deficiency and elevated CRP [15,18,19,21], while a statistical relation or data collection was lacking for the remainder. On the other hand, a clearer trend was found for the activity score in relation to zinc deficiency, although the scores used were heterogeneous across reports. However, available data are still insufficient to comprehensively analyze the prevalence categorized by the activity score variable, although this covariate is certainly to be considered in the fluctuations of the prevalence of zinc deficiency in IBD.

The limited data and biographical heterogeneity of the study populations reduce the reliability of this meta-analysis in qualitative terms. In addition, designs differed among the selected studies: the cross-sectional was the most common. However, this is the first meta-analytic study to be conducted on the prevalence of zinc deficiency in IBD. Further investigation is needed to corroborate and better define these data and how they may fluctuate in relation to disease activity.

## 5. Conclusions

Prevention of micronutrient deficiencies has the potential to reduce the risk of disease-related disability. Still, more evidence is needed to corroborate these first prevalence metadata. The present research highlights the importance of considering zinc as a micronutrient to be monitored, because every second IBD patient shows a deficiency. According to our results, zinc deficiency is more prevalent in the CD population, probably due to the more severely malabsorptive nature of this condition and also in light of the proximal site of zinc absorption in the intestine. In light of this, the latest ESPEN micronutrient guidelines [33] point out that the dietary reference intakes (DRI) of zinc for adults should be 8–15 mg. However, in malabsorption conditions, such as short bariatric surgery, cystic fibrosis, chronic pancreatitis, and IBD, the need for higher amounts of zinc (30–40 mg daily) to maintain the zinc balance needs to be considered.

## Figures and Tables

**Table 1:** Descriptive of studies selected from the literature screening process. (*n* = 9).

Authors, Year [Ref.]	Survey Year	Country	Study Design	Age (Years)		Sample Size		Sex (Female)		Serum Zinc Levels		Deficiency Cut-Off	Summary of Findings	Overall Risk of Bias
				**CD**	**UC**	**CD**	**UC**	**CD**	**UC**	**CD + UC**				
Ehrlich, Shay, et al., 2020 [15]	2000–2016	Asia (Israel)	Longitudinal, 7-year	14.1 (12–16) *	13.5 (10.8–15.7) *	225	38	96/225 (43%)	18/38 (46%)	70.5 ± 16.3 mcg/dL		≤70 mcg/dL	The prevalence of zinc deficiency in patients with CD at diagnosis was 88% (CD) and 31.6% (UC) in patients with IBD.	Low risk
				**CD + UC**		**CD**	**UC**	**CD + UC**		**CD+ UC**				
Han, Yoo Min, et al., 2017 [16]	2013–2015	Asia (Korea)	Cross-sectional	32 (16–70) ♯		34	49	19/83 (22.9%)		76.6 ± 14.9 mcg/dL		≤70 mcg/dL	Many Korean patients with IBD have zinc deficiencies, suggesting the need to monitor levels of these micronutrients.	Moderate risk
				**CD**	**UC**	**CD**	**UC**	**CD**	**UC**	**CD**	**UC**			
Macmaster, Damianopoulou, et al., 2021 [17]	2017–2018	Europe (UK)	Cross-sectional	48.0 (19.5–78.4) ♯	47.2 (21.0–78.5) ♯	59	30	37 (63%)	16 (53%)	NA		Laboratory range (not specified)	Zinc deficiencies had been found in 23.7% (CD) and 76.6% (UC) of subjects with IBD	Moderate risk
						**CD**	**UC**	**CD**	**UC**	**CD**	**UC**			
Schneider, Caviezel, et al., 2020 [18]	2016–2017	Europe (Switzerland)	Cross-sectional	41.32 (14.5) ‡	41.6 (13.7) ‡	98	56	48 (49%)	31 (55%)	NA		<10.7 µmol/L	In this study, insufficient serum zinc concentrations were observed in 11.2% of patients with CD and in 14.3% of patients with UC	Moderate risk
				**CD**	**UC**	**CD**	**UC**	**CD**	**UC**	**CD**	**UC**			
Siva, Rubin, et al., 2017 [19]	2000–2015	America (USA)	Longitudinal, 3-year	NA	NA	773	223	421/773 (54%)	107/223 (48%)	NA		<0.66 mg/mL	Patients with IBD with serum zinc deficiency are more likely to have adverse disease-specific outcomes	Low risk
				**CD**	**UC**	**CD**	**UC**	**CD**	**UC**	**CD**	**UC**			
Ishihara, Arai, et al., 2021 [20]	2018	Asia (Japan)	Cross-sectional	13 (4–16) ♯	11 (1–16) ♯	98	118	30/98 (31%)	53/118 (45%)	64 (33–124) μg/dL *	69 (41–177) μg/dL *	<70 μg/dL	Prevalence of zinc deficiency in pediatric patients with IBD was 60.2% (CD) and 37.3% (UC)	Low risk
				**CD**	**UC**	**CD**	**UC**	**CD**	**UC**	**CD**	**UC**			
Sakurai, Furukawa, et al., 2022 [21]	2017–2020	Asia (Japan)	Longitudinal, 20 weeks	39.5 (23–63) ♯	56.0 (28–87) ♯	276	206	NA		57.5 (31–74) μg/dL	63 (46–74) μg/dL	<80 μg/dL	Zinc deficiencies had been found in 86.2% (CD) and 50.7% (UC) of IBD subjects	Moderate risk
				**CD**		**CD**		**CD**		**CD**				
Soltani, Zahra, et al., 2021 [22]	2018–2019	Asia (Iran)	Cross-sectional	39.2 ± 13.4	42 ± 16.2	65		49 (75.4%)		86.2 ± 17.0 ng/dL		Laboratory range (not specified)	Zinc deficiency was observed in 21.5% of a CD sample	Moderate risk
				**CD**	**UC**	**CD**	**UC**	**CD**	**UC**	**CD**	**UC**			
Cho And Yang, 2018 [23]	2012–2016	Asia (Korea)	Cross-sectional	14.4 (5.0–17.4) ♯	14.2 (9.9–17.4) ♯	49	16	16/49 (33%)	9/16 (56%)	71.5 (32.0–105.0) μg/dL ♯	77.0 (55.0–106.0) μg/dL ♯	<70 μg/dL	Zinc deficiencywas found in 44.9% (CD) and 31.2% (UC) of IBD sample	Low risk

Abbreviations: CD (Crohn’s disease), UC (ulcerative colitis). * median (IQR), ♯ median (range), ‡ mean (standard deviation.

**Table 2:** Description of selected studies for meta-analysis.

n	Authors, Year	Disease	Deficiency Cases	Total Cases	Prevalence (%)	CI 95%
1	Ehrlich, Shay, et al., 2020 [15]	CD	198	225	88.00	0.83 to 0.92
		UC	12	38	31.58	0.18 to 0.49
2	Han, Yoo Min, et al., 2017 [16]	CD	19	34	55.88	0.56 to 0.73
		UC	13	49	26.53	0.15 to 0.41
3	MacMaster, Damianopoulou, et al., 2021 [17]	CD	14	59	23.73	0.14 to 0.37
		UC	23	30	76.67	0.58 to 0.90
4	Schneider, Caviezel, et al., 2020 [18]	CD	11	98	11.22	0.06 to 0.19
		UC	8	56	14.29	0.06 to 0.26
5	Siva, Rubin, et al., 2017 [19]	CD	326	773	42.17	0.39 to 0.46
		UC	86	223	38.57	0.32 to 0.45
6	Ishihara, Arai, et al., 2021 [20]	CD	59	98	60.20	0.50 to 0.70
		UC	44	118	37.29	0.29 to 0.47
7	Sakurai, Furukawa, et al., 2022 [21]	CD	238	276	86.23	0.82 to 0.90
		UC	140	276	50.72	0.45 to 0.57
8	Soltani, Zahra, et al., 2021 [22]	CD	14	65	21.54	0.12 to 0.33
9	Cho and Yang, 2018 [23]	CD	22	49	44.90	0.31 to 0.60
		UC	5	16	31.25	0.11 to 0.59

Abbreviations: CD (Crohn’s Disease), UC (Ulcerative Colitis), CI (Confidence Interval).

## Data Availability

The original contributions presented in the study are included in the article, further inquiries can be directed to the corresponding authors.
